# Analysis of Top-Down Perceptual Modulation Considering Eye Fixations Made on a Bistable Logo

**DOI:** 10.3390/jemr19010008

**Published:** 2026-01-14

**Authors:** Guillermo Rodríguez-Martínez, Juan Camilo Giraldo-Aristizábal

**Affiliations:** 1 Academic Area of Creativity, Faculty of Economic and Administrative Sciences, University Jorge Tadeo Lozano, Bogota 110311, Colombia; 2 Academic Area of Marketing and Innovation, School of Management, University EAFIT, Medellin 050001, Colombia; jcgiraldoa@eafit.edu.co

**Keywords:** bistable logos, brand communication, bottom-up perceptual processes, top-down modulation, bistable perception, ocular fixations

## Abstract

Within the framework of brand communication, several companies choose to use bistable logos. These types of logos fall within the mechanisms inherent to bistable perception, where the interpretation of the two possible percepts involved may depend on the areas being observed or on prior instructions given to the observer to search for a particular shape within the ambiguous image. Perceptual factors related to the stimulus and the areas of eye fixation are called bottom-up aspects. The information exogenous to the bistable stimulus that determines perception is called top-down modulation. In order to determine whether certain bottom-up perceptual modulation areas for the Toblerone bistable logo are related to the search for each percept previously modulated by a written instruction, an experimental task was carried out with 34 participants using a Tobii T-120 eye tracker device, manufactured by Tobii in Danderyd, Sweden. Seven bottom-up modulation clusters were analyzed for ocular responses manifested in two different top-down modulation conditions. The results show that for each of the percepts, some areas correspond to the textual information offered as a top-down modulator. It is concluded that for the perception of the Toblerone^®^ logo, some areas are related to each percept, and the unimodal top-down modulation mechanisms operate in certain areas, while others can be assumed to be parts of the logo that contribute to the recognition of the two percepts involved.

## 1. Introduction

### 1.1. Ambiguous Images and Bistable Logotypes: Psychological Issues Involved

**Figure 1:**
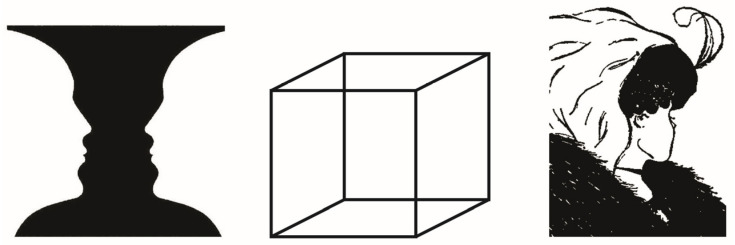
Examples of bistable images. On the (**left**), we present Rubin’s vase (an in-figure-ground reversal bistable image). In the (**middle**), there is Necker’s cube, an in-perspective reversal ambiguous image. On the (**right**), we show My Wife and My Mother-in-Law, an in-meaning content reversal image. Source: adapted from Rodríguez-Martínez and Castillo-Parra [4].

Within the context of brand communication and graphic design, the so-called bistable images have been used to convey different messages to audiences [1,2]. Based on the idea of being ambiguous figures [3], bistable images have been proven to be useful due to their capability of transmitting two different meanings, each one of them related to each possible visual percept [4]. When designing bistable logos, graphic designers know that it is not possible to recognize simultaneously the possible percepts that are involved within the ambiguous image [5]. As has been stated, during the observation of bistable images, there is no chance to perceive at the same time the two visual percepts [6]. Rather, just one percept remains recognized while the other one is not being perceived [7,8]. Once the observer starts to perceive the other possible percept, a perceptual reversal has taken place, that is, the switch from the first percept perceived to the second one [5,9,10,11,12,13]. Three examples of bistable images are depicted in Figure 1.

It has been noticeable how these images have been used in different fields: firstly, visual artistic images have been created based on the idea of provoking bistable perception [14]. In this regard, painters like Salvador Dalí or Archimboldo, painted images in which it is possible to alternate between two or more different visual percepts [15,16]. On the other hand, famous ambiguous images have been tested within the scope of the psychology of perception, such as the vase–face illusion [17], My Wife and My Mother-in-Law [18,19,20,21], the Schröder staircase [22], and Necker’s cube [23]. These kinds of tests have been conducted to prove that there are different perceptual mechanisms involved while looking at bistable images [4]. On the other hand, several studies concerning bistable perception have been carried out to analyze the observation of androgynous faces [24], since this kind of human images are considered both ambiguous and bistable [25]. Therefore, the perceptual psychological mechanisms that are involved in bistable visual perceptual processes apply to perceptual mechanisms linked to the recognition of the gender of androgynous human faces [24,26]. In addition, within the scope of brand communication, several brands and logotypes have been designed based on the principles that sustain bistable perception. Take, as examples, the logo of FedEx^®^ [27] and the logo designed for the brand Toblerone^®^ [28].

An issue that explains the bistable perceptual mechanism is the so-called bottom-up perceptual process that is involved due to areas that stand out influencing observers’ attention [5]. This way, physical characteristics of bistable stimuli exert an effect on their interpretation [29,30,31]. The realization of this effect depends on the areas of the image upon which observers’ eyes are fixed [1,8,18,19,32,33]. Nevertheless, expectations, concepts, and attitudinal predispositions play a role in the perceptual process as a whole [19,25]. When it occurs, the mechanism that is involved is a top-down modulating perceptual process [6]. In view of the foregoing, when information stored in memory and previous knowledge and expectations influence the final perception of bistable images, what is implied is the previously mentioned top-down perceptual process [5,34]. Thus, the interpretation of the ambiguous image is defined by information previously stored in memory [25,35] or also by inputs that enter into the visual perceptual system [36]. In this sense, when, for instance, auditory stimuli are influencing the perception of an ambiguous image, top-down perceptual modulation is operating [25], if the semantic load of the acoustic information is related to the meaning of the percept while observing a bistable visual stimulus [8,37]. Likewise, when an observer of a bistable stimulus reads a text whose content is related to one of the possible visual percepts involved, the semantic load of the text can influence the perception of the semantically congruent visual percept [38]. In that scenario, what emerges is the so-called semantic congruency effect [19,25].

A final issue that has to be mentioned to close this section is that, given the fact that bistable visual stimuli allow for two possible interpretations, they can also be called ambiguous images [39,40]. As mentioned before, when observers are looking at these kinds of images, the perception shifts between the two possible visual percepts that can be perceived [7,21]. These leaps are known as perceptual reversals [8,9,10,11,13,21].

### 1.2. The Conveyance of Semantic Load of Bistable Logotypes

The way consumers look at brands influences the potential conveyance of semantic loads [41]. If the logo itself is bistable, perceptual dynamics arise due to the likelihood of interpreting the visual design in two different ways [1,42]. Falling into the scope of bistable perception, logotypes that can be perceived in two different ways have the capability of conveying two different meanings, that is to say, two different interpretations [5]. The semantic loads concerning these interpretations normally go hand in hand in relation to concepts that are attributed to the semantic concepts of the brand involved [1]. It has been a matter of controversy whether all the consumers that constitute the target audience of a brand are able to identify the percepts that are involved in bistable logotypes, due to factors regarding visual gestalt configurations and figure-ground perceptual reversals [5,43]. That concern has also emerged based on psychological aspects regarding bistable perception, like bottom-up modulating perceptual mechanisms [8,44], as well as the areas of the logo on which ocular fixations are made [1]. In this spirit, when brand strategists decide to associate a brand with two different meanings that convey key concepts for that brand, visual designs usually emerge to communicate those brand values [45]. In this regard, graphic designers and advertisers design bistable logotypes, due to their potential to transmit two different meanings [1,27]. The question that arises here is related to the likelihood of the logotype being interpreted in such a way that the two percepts involved are recognized by consumers. If so, the two semantic loads involved for brand communication would be conveyed, which, in turn, would imply a complete communicative efficiency for the bistable logotype in question, based on Gestalt psychological principles regarding multi-stability [46,47].

As far as the logo of Toblerone^®^ is concerned, it is classified as a bistable image due to its capacity of offering two possible interpretations based on figure-ground visual perceptual principles. When looking at that visual advertising design, it is possible to recognize a mountain and, within the mountain, a bear standing on two legs [48]. According to classifications of bistable images, those based on the idea of disaggregating a form contained within another imply the possibility of perceptual bistability. Since the psychological perceptual principle of figure-ground operates, bistable images like the Toblerone^®^ logo are part of what are known as in-figure-ground reversals [4]. The fact that one figure is contained within the other, and that the contained figure contains essential features for the configuration of the other, fosters perceptual bistability, mediated by the figure-ground gestalt process [46], which is in turn manifested as a consequence of perceptual reversals [12]. This reaffirms the fact that processes of perceptual bistability involve gestalt processes that may involve the application of laws such as the law of closure or the figure-ground law [43,47]. The Toblerone^®^ logo contains two possible percepts, since when one is identified, the other (as a percept), although present in the visual field, is not in the foreground of Gestalt perception. A similar situation occurs with the FedEx^®^ logo, where the perceptible arrow defined by the contours of the space between the letters “E” and “X” is visible when viewing the entire logo, without any conscious perceptual recognition of it. This is why it is categorized as bistable, and where it is understood that there are not two simultaneous perceptions, but rather a transition between two possible different gestalt configurations [27], just as happens during the observation of the Toblerone logo^®^.

The history of Toblerone’s visual identifier (the logo itself) is intrinsically linked to its geographical origin. The brand was created in 1908 in Bern, Switzerland. Initially, the primary distinguishing element displayed on the box was the repeated triangular shapes of the chocolates, alluding to the Swiss Alps. At that time, the brand utilized a logotype designed by Emil Bauman, whose typeface was both simple and unique (the structure of this typeface is still preserved today). Later, in 1930, the brand sought to further connect with its country of origin [49] by including an illustration of an alpine bird perched atop the flags of Bern (featuring a bear) and Switzerland. In the late 1960s, the brand’s visual identity evolved into an imagotype, incorporating the Matterhorn, also known as Monte Cervino [50], one of the most iconic peaks in the Alps due to its triangular shape and a symbol of national pride [51]. This served to associate the brand with the renown of Swiss chocolate, the overall quality of the Helvetic industry, and to consolidate the triangular shape of the packaging and product, making this chocolate stand out within its market category [52]. The designer subtly included a bear in the isotype’s negative space, hidden among the mountain’s folds, a direct association with Bern, the ‘City of Bears’. In 2022, the manufacturer Mondelez International decided to shift part of its production to Slovakia. This decision meant the brand could no longer use its ‘Swiss-made’ label and had to transition from the distinctive Matterhorn silhouette to a generic mountain. In some countries, only the logotype is now used. Despite these changes, the brand remains a global symbol associated with travel, tradition, and luxury, demonstrating the power of effective brand management, visual identity, imagery, and shapes once they are positioned in consumers’ minds [53]. The study described in this article will take this bistable logotype as a reference, considered, in the scientific literature, as a bistable image of the in-meaning content reversals type. It is classified in this category because the two possible percepts share areas and lines, to the point that discriminating the figure-ground type is more complex to perform than in other types of ambiguous [4], such as those called in-figure-ground reversals images, like The vase–face illusion [54]. The essential purpose of the research described in the following pages was to analyze the potential bottom-up modulation effect that can be exerted by an instruction given to observers through a written text that directly states that what they are going to look at is either a bear or a mountain. In this sense, the texts ‘bear’ and ‘mountain’ operate as semantic perceptual modulators. It was hypothesized that certain areas of the Toblerone^®^ logo related to the visual percept associated with the semantic category of each text (bear and mountain) would be mostly observed. In this sense, as will be described later in the methodology section, critical bottom-up modulation clusters were preselected for the two possible visual percepts in question. Another objective pursued with the study was to establish the relevance of the critical areas of bottom-up modulation in relation to each of the visual percepts, understanding that the lines that define the figure of the bear are part of the mountain, which encourages a deeper investigation into the role played by the areas of fixation in relation to the detection of each of the figures (bear and mountain) involved in the brand design of a bistable nature.

## 2. Related Work

Various research studies have been conducted to establish the communicative effectiveness of a bistable logo. The assumption is that the two percepts involved in the design of a bistable logo must be recognizable by consumers and buyers [1]. This highlights the fact that these two possible percepts should have the same probability of being identified. However, scientific evidence shows that several factors influence the identification of the two visual percepts involved, such as salient areas of the logo [48], the way the observer looks at the brand identifier [55], eye fixations on the bistable design [19], and potential semantic modulators related to exogenous information the observer has in mind when observing the bistable design [56].

To begin with, there are studies that assess the areas of bistable logos that influence the likelihood for the two percepts involved to be recognized. In 2019, Rodríguez and Marroquín-Ciendúa sought to demonstrate that placing fixation points that direct the gaze toward certain areas of various bistable logos could have an effect on the way those logos are perceived. Indeed, upon reviewing the results, these researchers demonstrated that there are certain areas and visual clusters of bistable logos that, when observed, favor the recognition of each of the two visual percepts involved. Furthermore, upon analyzing the results of this study, it was found that critical zones of perceptual influence can be established, which, in the jargon of the psychology of visual perception, links to the previously mentioned bottom-up perceptual modulation [57]. These zones of influence effectively determine or direct perception as long as they are observed [18]. In this sense, what stands out is the importance of being able to observe, with eye-tracking devices, the areas where observers place their fixations when looking at a bistable image [3]. These areas can be grouped into clusters, that is, into areas that are defined by a set of fixations that, within a certain visual area of the logo in question, configure a surface, an area of bottom-up perceptual modulation [27].

Since the brain does not process visual information during saccadic movements [58], it is the eye fixations and their durations that contribute to the understanding of brand communication through the use of bistable logos [5]. It is also for this reason that there is an indivisible connection between technology and communication, where motion detection systems based on the recognition of oculomotor activity allow a deeper understanding of the perceptual mechanisms involved in the decoding of visual brand communication proposals [59].

Rodríguez-Martínez, in both 2024 and 2025, managed to demonstrate that there are bottom-up modulating areas that, as a matter of fact, seriously condition the identification of the percepts involved in a brand design of a bistable nature. In his first study [5], using an in-line design and eliminating color fills, he managed to demonstrate that for a bistable logo of a Colombian milk brand, there are areas that condition the perception and identification of a first percept, and that, likewise, there are other bottom-up modulation areas that favor the recognition of the second visual percept. This study, which was carried out thanks to the recording of the eye movements of the observers who voluntarily constituted the sample for the experimental tests, confirmed the psychological foundation of visual perception that defines the characteristics of the visual stimulus and the way in which these are observed through fixations as critical factors of the bottom-up type [60]. In this study, the bistable logo was displayed for 30 s.

In the second study carried out by this same researcher [1], the same paradigm of online logo exposure without color fill was taken into consideration, this time to identify areas of perceptual modulation in a logo with different characteristics than the first study, where the key was that the logo could have bottom-up modulation clusters dispersed throughout the design. In that study, the exposure to the bistable stimulus was also 30 s. Upon reviewing the results, it is concluded that, once again, there is perceptual conditioning mediated by the areas of the bistable design on which fixations are made [3]. These studies are joined by other research efforts that have sought to demonstrate aspects of bottom-up modulation that affect the observation of logos and brand emblems, depending on their position within a given advertising design. This is the case of the study developed by Girisken and Bulut [61]. In this spirit, there could be a kind of integration between semantic loads provided by the brand and advertising content derived from a creative concept [62]. For their part, Rodríguez and Marroquín-Ciendúa [27] used attention points to direct fixations on bottom-up modulating areas of bistable logotypes. It was demonstrated that manipulating attention to certain areas affects the interpretation of bistable brand identifiers by displaying visual stimuli for a period of 15 s.

On the other hand, Puškarević et al. [63], using eye-tracking technology, contrasted the attentional effects of brand fonts in advertisements. Oculomotor activity measures related to fixations showed the occurrence of bottom-up modulating effects, especially when brands use rhetorical language in conjunction with font types with a higher attentional load [5]. Another psychological factor involved and normally considered in this type of study is the level of attentional salience of the elements that constitute a certain bistable design [64]. In this sense, aspects of advertising visual stimuli and logos, such as the size and contrasts between communicative visual forms, can exert a significant influence on what consumers ultimately observe and identify. As has been stated, when observers look at labels or graphic designs where brands are present, a fact that is relevant for brand strategists is that it is very likely to differentiate them from competing products [5,65].

For their part, Tseng and Chuang [41] conducted a study focused on analyzing eye fixation behavior during observation processes of logos with illusory contours. Each logo display lasted 10 s. Assuming bistability, meaning that if perceptual closure of the parts where the illusory contour needs to be imagined is not performed, provoking ambiguity while trying to decode the graphic design itself, hidden contour logos were found to be more novel, interesting, and pleasing, which, according to the study, is in line with modern logo design trends. Furthermore, logos with ambiguous contours were also found to involve more dispersed vision vectors, along with longer fixation’s durations compared to text-type logos, which have a much more focused rather than dispersed distribution, based on sustained vision parameters. Nevertheless, the study suggests that too many missing parts in logos can affect their recognition [41], where a perceptual ambiguity emerges that leads to instability in the recognition of visual percepts necessary to convey brand semantic values [66].

Regarding the study of the semantic congruence effect and top-down modulation processes involved in the observation and semantic decoding of bistable logos, there are no studies focused on addressing these psycho-perceptual phenomena. However, the demonstration that semantic loads exogenous to bistable visual stimuli directly influence the perception of the visual percepts that correspond to the meanings of said external information has been widely documented in the scientific literature, e.g., [8,19,24,25,67,68]. Semantic correspondences, which underpin the concept of multisensory integration mediated by semantic congruences [69], can occur, first, in unimodal sensory modalities, where prior visual information, for example, influences the perception of a subsequently presented bistable visual stimulus [4], or in crossmodal modalities [70]. The latter implies that top-down modulating information comes from a sensory stimulation different from the sensory modality in which a bistable stimulus is presented [25]. Thus, acoustic information with a certain semantic load, for instance, can influence the recognition of a percept belonging to a bistable visual stimulus [8], where the semantic load of the auditory stimulus directs the recognition of the visual percept with a similar semantic load to the audio previously provided [24]. Here, the concept of perceptual semantic congruence emerges [19], where, as a matter of fact, the visual perception is conditioned by exogenous information but presented in a different sensory modality, which accounts for the multisensory integration phenomenon [71].

## 3. Method

### 3.1. Participants and Procedure

**Figure 2:**
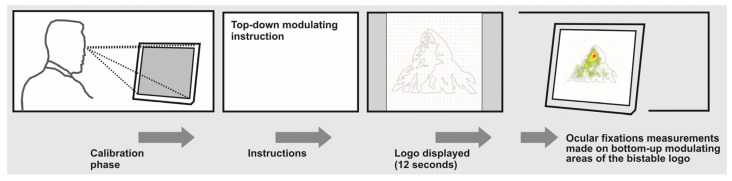
Diagram of the experimental task performed. Source: own design.

**Figure 3:**
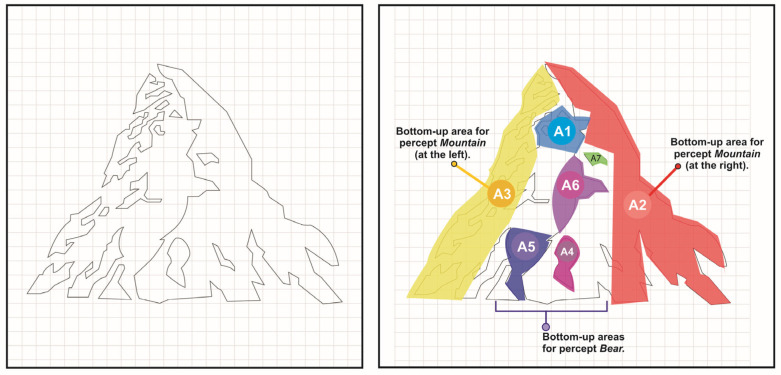
The bistable logo of Toblerone^®^ used for the study. On the (**left**), the outlined, unfilled version of the logo that was used. On the (**right**), the seven clusters or AOIs defined as bottom-up perceptual modulating areas. Zones A1, A4, A5, A6, and A7 were defined as bottom-up modulating areas for the ‘Bear’ percept, following the previous study conducted by Valderrama [48]. A2 and A3 areas (regarding the perception of the ‘Mountain’ percept) were also defined following the mentioned study. Source: adapted from Valderrama [48].

A total of 34 volunteers participated in the study (age mean, M = 22.41; SD = 2.58; women, 55.88%; men, 44.11%). Each of them continuously viewed the Toblerone^®^ logo in the simplified, unfilled, line version (see it in Figure 2 and Figure 3), following the analysis paradigm for recognizing bottom-up modulation areas for bistable images suggested in preliminary studies [1,5,18]. The bistable logo stimulus in question was displayed at two different times over a 12 s period on a Tobii^®^ T-120 reference eye tracker. This exposure time was established in consideration of what was performed in previous studies relating to the analysis of fixation areas during the observation of bistable logos, where the exposure times of the logos involve an observation that can range from 30 to 10 s, as found in previous studies previously documented in this article [1,5,27,41]. Regarding the size of the displayed logo, it was scaled to fit the screen. The displayed image was, as mentioned, the Toblerone^®^ outlined logo. It should be added that this logo was centered within a grid of 25 squares horizontally by 25.5 squares vertically, following the methodology implemented in previous eye-tracking studies on bistable logos drawn in a line [1,5]. The idea behind using the grid was to center the logo within it, scaling it until it reached the second or penultimate square in one of its dimensions. Once the logo image was obtained on the grid (see Figure 3, on the left), it was scaled to reach the limits of the eye-tracker device’s monitor, as can be seen in the descriptive image of the experimental task (Figure 2, logo displayed image).

All participants had to have normal vision or, failing that, corrected vision with contact lenses in order to observe the eye tracker monitor at a distance of 60 cm, following the protocols suggested by the Swedish manufacturer Tobii (Danderyd, Sweden) [64]. In this study, none of the participants wore glasses to correct vision defects. Each participant signed their informed consent, and the study was approved by the Ethics Committee of Jorge Tadeo Lozano University. The present study was conducted in accordance with the Declaration of Helsinki and approved by the Institutional Review Board of the University Jorge Tadeo Lozano (internal code 5414; approved on 5 August 2024).

Regarding technical specifications of the eye tracker monitor (Tobii T-120), its brightness complies with the European standard IEC/EN 60825-1/A1-A2, Class 1, for LED products intended for prolonged image exposures [72]. This standard establishes an acceptable emission limit to ensure continuous exposure, defined as the maximum illumination to which a person should be exposed for eight hours a day for several consecutive days. The suggested distance between the monitor and the observer, which is 60 cm, allows the time-averaged light exposure to be equivalent to 0.1% of the limit permitted for prolonged exposures, according to the regulations [64,72].

For each participant’s recording, a calibration phase was first performed, ensuring optimal recording of eye movements and ensuring an adequate sample rate. Participants were then given clear and precise instructions to keep their heads still and stare at the image to be presented. Since the visual stimulus was presented for 12 s, the need to remain highly focused during the two presentations of the bistable logo was emphasized. The two different presentation times corresponded to two different instructions, both of which involved top-down modulating information. This way, the two different conditions were defined by the use of a text that conditioned each participant to search for a specific percept within the displayed bistable image. Thus, the first instruction was: “Next, you will see a picture. Observe it carefully and identify the shape of a bear.” The second instruction was: “Next, you will see a picture. Observe it carefully and identify the shape of a mountain.” In this sense, the words ‘bear’ and ‘mountain’ constituted the exogenous semantic information that would influence the final perception of the bistable logo. In this way, it was expected that the hypothesis would be fulfilled that bottom-up areas corresponding to the percept related to the modulating word given in the instruction would be viewed more than visual areas unrelated to the modulated percept. The diagram regarding the experimental task is shown in Figure 2.

The two instructions were presented in a different order, using a randomization code inserted into the software setup of the eye tracker device used. Thus, the instruction to search for the ‘bear’ was sometimes the first instruction, or sometimes the second. The same situation occurred for the instruction to search for the ‘mountain’ visual percept. In this way, the order of presentation of the modulating information was counterbalanced to eliminate the bias associated with the order of the condition controlled by the researchers.

### 3.2. Data Analysis

All eye movement and fixation recordings were stored in Tobii Studio^®^ software, specifically the original platform for the T-120 hardware that does not have a specific version number (version of Tobii Pro Studio prior to 3.4.2.). For the purpose of analyzing fixations on critical bottom-up modulation areas, seven clusters or areas of interest (modulating areas for both ‘bear’ and ‘mountain’ percepts) were defined within Tobii Studio^®^ software. As shown in Figure 3, each of the areas of interest (AOIs, according to the convention used by the Swedish manufacturer Tobii^®^) was duly delineated and coded, assigning the codes A1, A2, A3, A4, A5, A6, and A7. Thus, the assigned codes have the following correspondences: A1, the bear’s face; A2, right side of the mountain; A3, left side of the mountain; A4, the bear’s lower right paw; A5, the bear’s lower left paw; A6, the bear’s upper left paw; A7, the bear’s upper right paw. The designation of the bear’s paws as left or right was assumed based on the observer of the logo and not based on the bear itself. These corresponding clusters (AOIs) are depicted in Figure 3.

Once the areas of interest were defined, ocular metrics referring to the time of fixations involved in each of the seven clusters were extracted. The areas of interest were duly selected following the protocols previously designed by Bernal-Robayo [73] and subsequently considered and implemented in the studies carried out with eye-tracking techniques by Rodríguez-Martínez [1,5]. The corresponding analyses were performed for each of the two top-down modulation conditions, and the relationships between the observed areas and their equivalence with the modulating text given in the instructions were established. Tobii Studio^®^, Excel^®^ (v. 16.45), and SPSS^®^ (v. 23) were used for the corresponding statistical analyses.

## 4. Results

**Figure 4:**
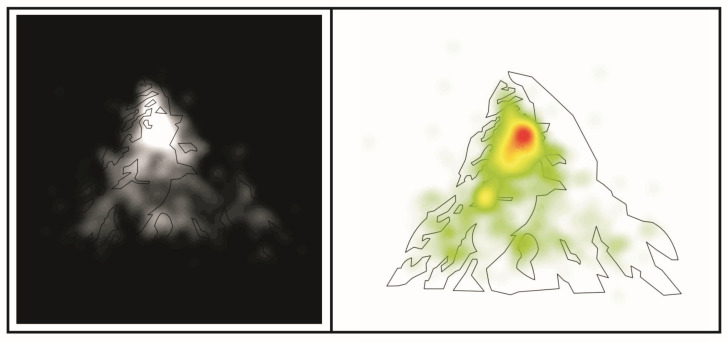
Two eye-movement maps concerning all the ocular data captured, meaning the totality of data resulting from bringing together all the fixations manifested in the two top-down modulation conditions. On the (**left**), an opacity map in which the brighter the area, the more it was viewed. On the (**right**), the corresponding heat map. Source: own design.

In this section, we consider all the results obtained after analyzing all the durations of fixations made in the selected AOIs. The ocular metric referring to the duration of fixations is very relevant to determine the degree of importance and attentional salience of a certain area within the observer’s visual field [74]. Understanding that the visual field is established by an area defined by both horizontal and vertical vectors (horizontal horopter and vertical horopter), it is just in the center where the best-focused area is established [75], which refers to the place where the observer is looking (ocular fixation) when a saccadic movement has stopped [58]. In Figure 4, the totality of the ocular data referring to fixations is graphically depicted by means of two ocular maps: an opacity map (on the left) and a heat map (on the right). On a heat map, the areas depicted in red are the most gazed upon areas [74]. These images will be discussed later.

Starting with cluster A1 (the bear’s face), no statistically significant differences were found in favor of the moment at which observers reviewed the image with the modulating text referring to the bear concept. While the difference between the means of the durations of the fixations did favor when observers read the instruction about searching for the bear (M = 2.45, SD = 1.34) compared to when the image was viewed with the conditioning of searching for the mountain (M = 2.17, SD = 1.19), the statistic did not show a value that suggested a significant difference (t(33) = 0.99; *p* = 0.32). It is possible that there was a slight impact on perception mediated by the instruction, but without any marked difference being found in the compared averages of the durations of the fixations manifested in that area. Regarding AOIs A2 (mountain area to the observer’s right), the result is in line with the hypothesis, since the average duration of fixations with the textual modulator referring to ‘mountain’ is significantly longer compared to the time when the observers were instructed to search for the bear (in the modulating text condition congruent with the mountain percept, M = 1.37, SD = 1.19; in the modulating condition for the bear percept, M = 0.70, SD = 0.51; t(33) = 3.25; *p* = 0.002). Clearly, a greater amount of observation is recognized in the mountain area to the observer’s right when there is congruence with the modulating text (*p* < 0.05). But contrary to expectations, in the case of the mountain area located to the left of the visual field (area A3), there is greater visual tracking (and with a statistically significant difference) in that area of the bistable logo when the instruction to search for the bear is given, compared to when the instruction orients the visual task towards searching for the mountain: in the modulating text condition congruent with the mountain percept, M = 1.97, SD = 1.27; in the modulating condition for the bear percept, M = 2.52, SD = 1.11; t(33) = 2.15; *p* = 0.03. A possible explanation for this phenomenon may be given by the proximity of that area of the mountain in relation to the body of the bear, as will be discussed later.

Turning to the specific analysis of the bear’s paw areas (one area for each paw, A4, A5, A6, A7), a review of the eye fixation times reveals that one of the areas of the two lower paws turns out to be key for bear recognition (area A5, which is further left in the visual field). According to the results, when observers were instructed to search for the bear, the cluster A5 had greater observation in terms of fixation durations, giving a significant difference in favor compared to the modulation given by the instruction referring to the search for the mountain. For the case of AOIs A4 (the bear’s lower paw on the right of the visual field), the t-student test statistic is, t(33) = 0.25, *p* = 0.79; mean value for top-down modulation in favor of the bear percept, M = 0.32, SD = 0.27; mean value for top-down modulation in favor of the mountain percept, M = 0.3, SD = 0.28. On the other hand, the data for the analysis of area A5 (the bear’s lower paw, left area of the observer’s visual field) are as follows: in the condition of congruent modulating text with the bear percept, M = 0.23, SD = 0.24; in the modulating condition for the mountain percept, M = 0.09, SD = 0.19; t(33) = 2.48; *p* = 0.01. As previously stated, this bear’s paw on the left side of the visual field is more frequently observed in the congruent top-down modulation condition, with a difference recognized as statistically significant (*p* < 0.05).

**Figure 5:**
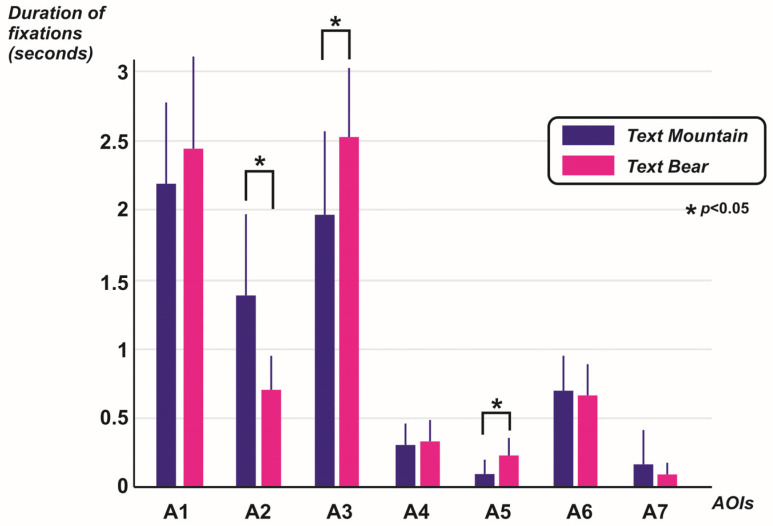
Comparisons of the average durations of fixations made in each AOI, taking into account the two top-down modulation conditions. Source: own design.

With regard to the bear’s upper paws (areas A6 and A7), no differences were found in the average durations of fixations when comparing the two top-down modulation conditions in either case. For the analysis of area A6 (the bear’s right paw, with a larger area or surface area than the other upper paw), the results of the statistical test were as follows: t(33) = 0.33, *p* = 0.73, where the average values were, for the mountain percept modulation condition, M = 0.69, SD = 0.46, and for the instructional text condition referring to the search for the bear, M = 0.66, SD = 0.43. Regarding area A7 (upper left paw of the bear), the statistic did not show a significant difference: t(33) = 0.84, *p* = 0.4, where the average values were, for the modulation condition for the mountain percept, M = 0.16, SD = 0.48, and for the instructional text condition regarding the search for the bear, M = 0.09, SD = 0.16. The comparisons between the average of the durations of fixations in each cluster and considering the two top-down modulation conditions are represented in Figure 5.

In Table 1, all the results are depicted, implicating all the AOIs taken into account and the results in terms of the means of durations of fixations made on bottom-up critical areas considering the top-down modulating texts.

## 5. Discussion and Practical Implications

There is a relationship of semantic congruence in relation to some of the bottom-up modulating areas, as occurs in areas A2, A3, and A5. Taking into account that the paradigm being used is that of the correspondence between a greater number of durations of fixations in areas pre-valued as favoring the recognition of the percept previously modulated by the information given in the instruction [48], it is seen that areas A2 (favoring the mountain percept) and A5 (favoring the bear percept) achieve the expected correspondence effect. This is because, in the case of area A2, fixation durations are in fact longer for the condition in which the text refers to the expected visual percept, the mountain. The same applies to area A5 (the leftmost paw of the bear in the visual field): there is also a significant difference in favor of the duration of fixations in the top-down modulation condition defined by the instruction to search for the bear. A first consideration to estimate is the fact that area A3, preliminarily assessed as an area favoring the mountain percept [48,73], had the highest level in terms of duration of fixations in the modulating condition for the bear percept (M = 2.52, SD = 1.11), compared to the durations of fixations referred to the top-down modulation condition corresponding to the search for the coherent percept (mountain) in terms of bottom-up fixation areas (M = 1.97, SD = 1.27). This difference turned out to be significant (*p* < 0.05). Two considerations could eventually explain this result: first, that some boundaries of area A3 are very close to the contour lines that define a part of the bear percept. Compared to area A2, which would also contribute to the recognition of the mountain, this has a significant surface area that is far from the shape of the bear, sharing a smaller amount of boundary contours with the posterior area of the bear percept. Ambiguities in relation to contours can often impact the perceptual performance of observers [41]. The second consideration to take into account is that, possibly due to the location of area A3 within the visual field (on the left and covering almost the entire verticality of the bistable visual stimulus), observers fix their gaze there more due to a cultural behavioral habit marked by the fact that in Western cultures people learn to read and observe starting from the left and from top to bottom [76]. Since the present study did not look at saccadic movements, their vector path and displacement pattern, the above explanation remains a supposition, which merits further research that elucidates the phenomenon based on visual path patterns [58] and not only on the basis of analysis of fixations. This implication is not minor, considering that other types of perceptual modulations are related to habits, learned behaviors and observation patterns based on the path of saccadic movements [77].

On the other hand, the difference with which a very clear correspondence is established between the top-down and bottom-up levels in relation to area A2 (which, according to previous studies, is an area that favors the recognition of the mountain percept) is notable. As a matter of fact, the greater amount in the duration of fixations manifested there when there is correspondence with the textual modulator (M = 1.37; SD = 1.19) turns out to be significant (*p* < 0.05) when contrasted with the permanence of fixations in the opposite top-down modulation condition (M = 0.7; SD = 0.51). According to the results, we have acquired greater empirical evidence that this area is associated with the recognition of the mountain percept. However, understanding that during visual recognition tasks under bistable perception paradigms, stochastic-type behaviors are recognized [4], eye visits will normally be found throughout the visual field, regardless of the attentional modulations involved [78]. Again, there is a need to advance future studies where multiple variables and co-variates involved are controlled, to the point that it is possible to recognize the level of bottom-up modulation of some areas of visual stimuli, and the incidence that exogenous information may have at the level of oculomotor activity that conditions perception in terms of top-down modulations. In this sense, it is also considered that the way in which the attempt is made to condition perception from top-down mechanisms has an echo in the perceptual effects involved in bistable perception paradigms [4]. It is not the same to exercise perceptual modulation through a text placed previously to the exposure of a bistable visual stimulus, than to do so through a modulating audio that is constantly present during the exposure of the ambiguous image [8,20,24]. The way in which modulating exogenous information enters the memory system before or during the perceptual recognition process affects both cognitive and perceptual performance. In addition, factors associated with the workload involved through the use of working memory [79,80], or through the use of long-term memory, have an indisputable impact on the perceptual modulation process and the corresponding fixations and derived saccadic movements [58].

Moving on to another result worth considering, it is important to refer to the visual processing given to area A1 (the bear’s face). Considered an essential area for bear recognition [48], and given that its position within the visual field is relatively privileged (almost in the center of the figure and at the top, somewhat close to a threshold point with relevant attentional salience such as the mountain peak), this area is highly observed and has significant durations of fixations there, as demonstrated in both top-down modulation conditions. According to the results, fixation durations do increase when the instruction to search for the bear figure is given, although the difference is not statistically significant: M = 2.45, SD = 1.34, compared to the conditioning of searching for the mountain, M = 2.17, SD = 1.19. It is worth highlighting here that there may be a potential neutral modulation area (A1), that is, the area itself can contribute to the recognition of the two percepts involved in the bistable image [18,19]. Nevertheless, it must be taken into account that areas located in central regions of a bistable visual stimulus may be favored in terms of their probability of being observed, since, due to coordinates and location within the visual field, it is less demanding for the observer to focus on that area [19,27]. Additionally, due to developmental issues related to learned behaviors and visual performance, images and shapes that resemble faces of living beings have a level of salience that gives them a certain probability of being observed when they are part of a bistable visual stimulus [3]. In this sense, it becomes clear that forms recognizable through matching with stored memory exert an attentional effect that conditions the perceptual interpretation of bistable stimuli [4,7]. In this vein, future studies interested in uncovering the modulating mechanisms involved in bistable perception should analyze patterns and behaviors that condition perception and that can encourage the recognition of one or the other percept, when the area itself and what it visually represents can contribute to the recognition of both possible percepts. Thus, neutral modulation areas can be identified [8,18] that may nevertheless be influenced to favor a particular percept through top-down modulation [20]. Coming back to the AOIs A1, and according to the results, there would be evidence to recognize a minimal impact from the top-down modulator used. In this regard, it is also suggested that future studies be conducted to analyze the bottom-up conditioning effect for this and other areas, not only in this bistable stimulus, but also in others where the same area is relevant for the perceptual interpretation of the two percepts involved. This phenomenon of areas favoring both possible percepts is particularly evident in in-meaning-content reversal bistable images [4], given that the two percepts share not only contour areas but also shapes and surfaces that are relevant to the configuration of the two potential gestalts involved [18,43]. This consideration applies to other areas involved here (the Toblerone^®^ logo) and to any study that seeks to determine bottom-up modulation conditions based on the orientation of perception toward disambiguation in the interpretation of bistable stimuli. The preliminary study by Valderrama [48] had already suggested that area A1 is highly relevant to the understanding and perceptual decoding of this logo, as this part of the design contributes to the recognition of the bear, but it is also a key part of the upper-central part of the mountain.

Turning to the analysis of the bear’s paws, it is noteworthy that area A6 has a greater number of eye fixation durations compared to other areas of the bear’s paws, such as A4, A5, and A7. Area A6 is one of the bear’s paws, very important for recognizing the animal involved in the image [48], since it is the bear’s right front paw and covers a larger area than the other upper paw (A7) and the bear’s lower left paw (A4). Neither areas A4, nor A6, nor A7 showed a behavior where the correspondence between their durations in terms of fixations and the corresponding top-down modulating condition could be recognized. Returning to area A6, although it stands out slightly (see Figure 4) in relation to the other 3 areas corresponding to the bear’s paws, it is not possible to mention the possibility of a top-down modulating effect when comparing the average durations of fixations in the visual correspondence areas (*p* > 0.05). In fact, and as previously mentioned, of the four paws of the bear, it is only the one referring to area A5 (lower right paw of the bear) that involves the correspondence between the top-down and bottom-up levels involved (regarding the condition of congruent modulating text with the bear, M = 0.23, SD = 0.24; in the modulating condition for the mountain, M = 0.09, SD = 0.19, *p* < 0.05). It is worth mentioning that, when reviewing both the opacity map and the heat map in relation to fixations (Figure 4), it becomes evident (descriptively speaking) how there is a concentration of fixations in area A1, but understanding that, due to its surface area (smaller than that of A3), surface A3 is also visited, but with a greater dispersion of fixations throughout it (as previously stated, of larger surface area). The opacity map (on the left of Figure 4) shows what observers actually see when looking at the Toblerone^®^ logo, showing how the triangle that is the basis of the design of this logo is configured, but where there are definitely areas that are mostly observed. It has to be kept in mind that these maps cover the entire captured data, without distinguishing between observations in one or another top-down modulation condition. In addition, it should be noted that, in order to minimize errors in the comparisons made, the fixations observed under the two top-down modulation conditions were compared, considering previously defined bottom-up areas from a prior exploratory study. Each area corresponds more closely to each of the percepts involved, leading to the hypothesis that exogenous modulating information should have a greater effect on the duration of eye fixations in the areas of the percept being searched for via top-down modulating instruction. It should also be considered that, given that the logo under study is of the in-figure-ground reversal type, and that it also has one figure within another, there could inevitably be areas that can be fixed for the perception of one or the other percept. This fact highlights the presence of so-called neutral modulation zones, that is, areas of the visual design that can function, in terms of the physical characteristics of the stimulus, as indistinct bottom-up modulators. Further studies can be performed in which participants are required to report what they are recognizing in the image, recording the areas where fixations are being made, seeking to demonstrate that, for certain bistable images, there will be areas that can equally (or at least at a similar level) favor the recognition of the two percepts involved.

To deepen and enhance the scientific approach to bottom-up and top-down mechanisms operating in perceptual bistability processes, it is essential to consider that the present study has several limitations and that future studies could include improvements. First, a larger sample size should be used to make the results more generalizable. Second, experimental studies should be proposed that compare one or more bistable logos not only with contour lines but also in their original versions with colors and fills. Another recommendation for future research is to review the potential influence of the exposure time of the bistable logo, naturally taking into account the purposes of the study. As previously mentioned, the exposure time chosen for this study was assumed to be within ranges previously implemented in studies related to the analysis of modulated perceptual bistability during the observation of bistable logos. On the other hand, showing bistable logotypes within real advertising contexts would help to recognize visual perceptual processes regarding bistable perception, where, as an example, the information provided by the ad could operate as a top-down perceptual modulator. Additionally, design features can be manipulated to further explore bottom-up modulation, going beyond simply comparing these mechanisms by observing fixations in critical areas of bottom-up modulation pre-validated in previous studies. Bistable logos of different types can also be compared (e.g., in-perspective reversals or in-meaning-content reversals). Likewise, future research may consider other oculometry measurements, such as saccade trajectories, eye fixation counts, scanning patterns, etc. It is also recommended to conduct a new study in which the hypothesis is to determine the most salient areas at the attentional level, without top-down modulation, in order to establish stimulus prominences that condition the search for a percept when it is modulated by information exogenous to the bistable stimulus itself.

One final consideration should be made regarding future studies based on the statistical analysis strategy. In the present study, paired analyses were performed for each pre-selected bottom-up modulation area, comparing the durations of fixations in each area according to the two bottom-up modulation conditions. In this sense, seven different comparative analyses were generated, one for each bottom-up modulation area. In the future, seeking to avoid potential errors or biases, experimental designs could be implemented that establish conditions in a factorial manner. This would strengthen the approach to addressing the influence of top-down modulation in certain areas, but by using repeated measures or generating a factorial design to obtain greater precision regarding the relationship between the exogenous modulating information and the observational areas involved in the detection of the percept previously modulated through instruction.

To close this section, it should be pointed out that the results discussed here should be taken into account by graphic designers and brand communication strategists, since brand logos and emblems are essential for communicating values and semantic implications that contribute to a brand’s strategic positioning. When dealing with bistable logos, it is essential to consider the psychological aspects involved in the perceptual interpretation process. Thus, designers must understand that there are attentional saliences that condition or modulate (in a bottom-up way) bistable perception. They must also understand that in practical brand communication, contexts, information from advertising, or from the audience’s knowledge concerning brands, can be mixed [81,82]. This exogenous information can operate as a top-down modulator, echoing in the decoding of the corresponding bistable logo.

## 6. Conclusions

It is concluded that, for the Toblerone^®^ logo, there are certain bottom-up modulation areas that have a perceptual correspondence with a textual modulator presented in a unimodal sensory modality. In addition, it is recognized that the two possible percepts involved in this bistable logo may have corresponding critical areas of eye fixations that can contribute to the decoding of the image itself. From the analysis of the duration of fixations, there is an area of the bear percept (the bear’s lower right paw) that allows for top-down modulation and enables its interpretation. For the mountain percept, the entire right-hand area of the logo, from top to bottom, which constitutes an essential part of the mountain’s shape, is favorable for the recognition of that percept, presenting a correspondence between the top-down and bottom-up factors considered in the study. The right-hand area of the mountain has a proximity to the bear’s shape. This may be one reason why the top-down modulator operated in reverse, that is, there was a longer duration of fixations in that area under the modulating condition supporting the bear percept. The bear’s face area obtained a significant level of attentional salience, but without demonstrating a real top-down modulation effect. This study demonstrates the complexity involved in decoding bistable logos and suggests that designers and researchers in this field should delve deeper into the psychological factors involved in the decoding and interpretation processes of bistable visual stimuli used in the context of brand communication.

## Figures and Tables

**Table 1:** Statistical values concerning durations of fixations made on the AOIs.

AOIs	Statistical Data	Text Mountain	Text Bear	*p*-Value
A1	Mean	2.17	2.45	0.32
A1	Standard deviation	1.19	1.34	
A1	Variance	1.41	1.82	
A2	Mean	1.37	0.7	0.002
A2	Standard deviation	1.19	0.51	
A2	Variance	1.42	0.26	
A3	Mean	1.97	2.52	0.03
A3	Standard deviation	1.27	1.11	
A3	Variance	1.61	1.24	
A4	Mean	0.3	0.32	0.79
A4	Standard deviation	0.28	0.27	
A4	Variance	0.08	0.07	
A5	Mean	0.09	0.23	0.01
A5	Standard deviation	0.19	0.24	
A5	Variance	0.03	0.06	
A6	Mean	0.69	0.66	0.73
A6	Standard deviation	0.46	0.43	
A6	Variance	0.21	0.18	
A7	Mean	0.16	0.09	0.4
A7	Standard deviation	0.48	0.16	
A7	Variance	0.23	0.02	

## Data Availability

The datasets presented in this article are not readily available due to privacy restrictions. Requests to access the datasets should be directed to the corresponding author.
